# Rivaroxaban for cancer-associated venous thromboembolism: A systematic review and meta-analysis protocol

**DOI:** 10.1097/MD.0000000000018087

**Published:** 2019-11-27

**Authors:** Bo Liang, Li-Zhi Zhao, Hui-Ling Liao, Ning Gu

**Affiliations:** a Nanjing University of Chinese Medicine, Nanjing; b Hospital (T.C.M.) Affiliated to Southwest Medical University; c College of Integrated Traditional Chinese and Western Medicine, Southwest Medical University, Luzhou; d Nanjing Hospital of Chinese Medicine Affiliated to Nanjing University of Chinese Medicine, Nanjing, China.

**Keywords:** cancer-associated venous thromboembolism, protocol, rivaroxaban

## Abstract

**Background:**

All cancers increase developing venous thromboembolism risk, and VTE is the second-leading cause of death among cancer patients. The anticoagulant drugs are considered to be the optimal treatment for patients with cancer-associated VTE. However, there is still controversy whether rivaroxaban, a new oral anticoagulant, can lead to better outcomes globally.

**Methods:**

We will search PubMed, Web of Science, Cochrane Central Register of Controlled Trials and China National Knowledge Infrastructure for relevant published studies before 1 September, 2019, without any language restrictions. Only published randomized controlled trials that meet the inclusion criteria will be included. Subgroup analysis of the type of cancer, the type of VTE, cancer stage, age, sex, ethnicity, history of smoking and drinking as well as the mean, dose and duration of anticoagulants will be performed.

**Discussion:**

Our study aims to estimate the efficacy and safety of rivaroxaban for patients with cancer-associated VTE and to provide recommendations to key stakeholders.

**Trial registration:**

PROSPERO, October 23, 2019, CRD42019143265, https://www.crd.york.ac.uk/PROSPERO/display_record.php?RecordID=143265.

## 1 Introduction

Venous thromboembolism (VTE) refers to a condition in which the blood clots from a vein inappropriately, causing considerable morbidity, mortality and economic burden.^[1,2]^ It is the third leading vascular diagnosis after heart attack and stroke, affecting, to be estimated, between 300,000 to 600,000 Americans each year.^[1]^ All cancers increase developing VTE risk, especially if the cancer has spread widely, and if it is cancer of the lung, brain, lymphoma, gynecologic system, or gastrointestinal tract, and thrombosis is the second-leading cause of death among cancer patients because of activation of coagulation, use of long-term central venous catheter, thrombogenic effects of chemotherapy and anti-angiogenic drugs.^[3]^ Cancer-associated VTE is a prevalent and life-threatening complication in individuals with cancer,^[4,5]^ with clinically higher morbidity and mortality,^[6]^ and the complexity of its prevention and treatment is due to the much higher risk of complications, including recurrent VTE and major bleeding, in those patients than others.^[7,8]^ Medicines that help prevent further blood clots from forming or that dissolve serious vein blockages are the main treatments for VTE besides catheter-assisted thrombus removal and vena cava filter, including anticoagulants, or blood thinners, and thrombolytics. The management of anticoagulant therapy for the treatment of VTE in patients either with a diagnosis of cancer or in whom cancer is clinically suspected has also become a major concern among clinicians and relevant patients, since clinicians should consider the bleeding risk, the type of cancer, and the potential for drug-drug interactions in addition to informed patient preference in determining the most appropriate treatment.^[7,9]^ The National Comprehensive Cancer Network Clinical Practice Guidelines in Oncology for Cancer-Associated VTE outline strategies to prevent and treat cancer-associated VTE, however, it does not directly point out which medicine benefit more.^[10]^ Rivaroxaban is an anticoagulant and the first orally active direct factor Xa inhibitor.^[11]^ Although the FDA approved rivaroxaban to treat VTE based on clinical trials that patients are not fully with cancer,^[12–15]^ a subgroup analysis of cancer patients has been performed for these pivotal clinical trials,^[16]^ and randomized controlled trials specifically for cancer patients are currently available.^[17]^ Results about the safety and efficacy of rivaroxaban for cancer-associated VTE remain controversial, thus, we will conduct a systematic review and meta-analysis to estimate the efficacy and safety of rivaroxaban for patients with cancer-associated VTE and to provide recommendations to clinicians and patients.

## 2 Methods

This protocol adheres to the Preferred Reporting Items for Systematic Review and Meta-Analysis Protocols statement^[18]^ and the results of this study will be published and shared in an international peer-reviewed journal with reference to the PRISMA guidelines.^[19]^ Ethical approval and patient consent are not required as this study is based on published studies.

### 2.1 Studies search and selection

Two reviewers (BL and HLL) will search PubMed, Web of Science, Cochrane Central Register of Controlled Trials and China National Knowledge Infrastructure for relevant published studies before 1 September, 2019, without any language restrictions. The subject terms and keywords corresponding to Medical Subject Heading terms will be used to search for eligible studies in the databases as mentioned above. Search strategies in PubMed are shown in Table 1.

**Table 1:**
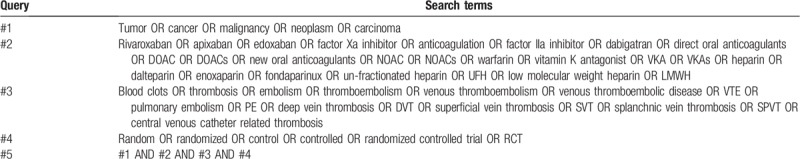
PubMed search strategies.

We will adopt the methods from the Cochrane Handbook for Systematic Reviews of Interventions to pool the evidence.^[20]^ Eligibility criteria for studies to be included in this study will be reported following the PICOS scheme^[21]^ in Table 2. The participants will be patients diagnosed with cancer-associated VTE regardless of the type of cancer, stage, sex, ethnicity, economic status or education. All anticoagulants for participants will be studied. The primary outcomes are defined as recurrent VTE and adverse bleeding events. The secondary outcomes are defined as the quality of life, complication rate and all-cause mortality. Only randomized controlled trials will be included.

**Table 2:**
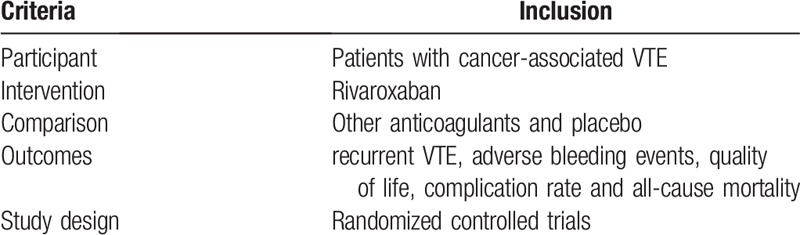
Eligibility criteria following the PICOS scheme.

All duplicate searched studies will be removed via EndNote X8 software before 2 reviewers (BL and HLL) independently screen each title and abstract. The full texts of all the possibly eligible studies will be checked independently and in duplicate by the 2 reviewers (BL and HLL). In addition, only the studies that do meet the inclusion criteria described above will be included, neither of the 2 reviewers will be blind to the reasons for exclusion. A third author (LZZ) will make final decisions in contradictory judgements. The fourth reviewer (NG) will oversee the entire studies screening and selection process to ensure the completeness and accuracy. The whole process will be shown in a PRISMA flow chart in detail.

### 2.2 Data extraction and management

One reviewer (BL) will extract the following data from remaining studies: the information about study characteristics (such as the first author, publication date, country, study design and methodology, follow-up duration, withdrawals and the period of the study), demographic characteristics (including age, sex, ethnicity, history of smoking and drinking, pathology diagnosis or clinical suspect and cancer stage), interventions (types and doses of anticoagulants where applicable) as well as outcomes. If the studies mention other drugs used, this information is also recorded for subsequent drug-drug interaction analysis. For missing data, we will contact the study investigators via email to obtain as complete information as possible. Then another 2 reviewers (LZZ and NG) will check the received data. The final author (HLL) will make final decisions in contradictory cases. Finally, all extracted data will be stored in the predesigned excel spreadsheet.

### 2.3 Quality assessment

Selected studies will be critically and independently assessed by 2 reviewers (BL and HLL) using a revised Cochrane risk of bias tool for randomized trials---RoB 2 tool.^[22,23]^ The assessments include methods of randomization, treatment allocation, blinding, and other biases.^[24]^ The third author (LZZ) will decide in the case of contradictory assessments of the 2 reviewers (BL and HLL) and the risk of bias in each domain will be assessed as high, low or uncertain, and the results of the evaluation will be shown on the risk of bias graph.

### 2.4 Data synthesis and analysis

The Review Manager 5.3^[25]^ and Stata 12.0 will be adopted to synthesis and analysis data. The results are likely to be substantially heterogeneous and will be synthesized according to the data type and following Centre for Reviews and Dissemination's guidance.^[21]^ If the data are highly homogeneous, a meta-analysis will be directly conducted with fixed-effect model. Otherwise, after the random-effect model, subgroup analysis will be performed, if the available data are enough, to identify which study the heterogeneity potentially came from. Depending on the type of extracted data, we may apply subgroup analysis by the type of cancer, the type of VTE, cancer stage, age, sex, ethnicity, history of smoking and drinking as well as the mean, dose and duration of anticoagulants. Moreover, in order to reduce or even eliminate the impact of heterogeneity, we will additionally conduct meta-regression and sensitivity analysis. Lastly, the funnel plot and Egger test will be implemented to assess for publication bias.

## 3 Discussion

To our knowledge, this study will be the first to focus on rivaroxaban on the treatment of cancer-associated VTE. In this study, we combined National Comprehensive Cancer Network,^[10]^ Canadian^[7]^ and French^[26]^ guidelines to broadly define VTE as deep venous thrombosis, pulmonary embolism, central venous catheter related thrombosis, superficial vein thrombosis, and splanchnic vein thrombosis. In recent years, many clinical trials about rivaroxaban and VTE in cancer patients have been carried out. Thus, we can more comprehensively evaluate the safety and efficacy of rivaroxaban on the treatment of cancer-associated VTE. As a therapeutic oral anticoagulant, rivaroxaban is mainly metabolized by CYP3A4 and serves as the substrate of the transporter protein p-glycoprotein. The incidence of bleeding complications or treatment failure is lower than that of traditional drugs, warfarin or other vitamin K antagonists. This study will also compare the 2. In addition, rivaroxaban has relatively few drug or food interactions, and we will also analyze if the data are sufficient.

This protocol was developed using validated tools and processes, yet, there are several noteworthy limitations. First, we might miss studies that are relevant to this review. We strive to minimize this issue by working with a librarian on the search strategy. Second, since many studies do not mention cost, it may be difficult to make an economic assessment.

Nevertheless, we anticipate that this study may highlight gaps in knowledge and provide insights into the treatments for cancer-associated VTE. These findings will also inform future research and key stakeholders in developing interventions more effectively and safely.

## Acknowledgments

The many relevant published studies are acknowledged for their support.

## Author contributions

BL, HLL and LZZ designed and planned the study. BL and NG wrote the report. All authors have read and approved the final version of the report.
